# Breaking boundaries: A tale of one of the longest ECMO support on the road to lung transplant

**DOI:** 10.5339/qmj.2024.qitc.8

**Published:** 2024-03-24

**Authors:** Irfan Ul Haq, Mansoor Hameed, Merlin Thomas, Takahiro Oto, Wael Khalaf, Tasleem Raza, Hisham A. Abdul Sattar

**Affiliations:** 1 Pulmonary Medicine Department, Hamad General Hospital, Doha, Qatar Email: ihaq@hamad.qa; 3 Surgical Intensive Care Unit, Hamad General Hospital, Doha, Qatar

**Keywords:** Lung transplant, ECMO, bronchiolitis, rehabilitation

## Introduction

The use of extracorporeal membrane oxygenation (ECMO) as a bridge to lung transplant has become more prevalent, enabling the stabilization of critically ill patients while awaiting transplantation. Typically, individuals on ECMO for under 30 days are considered eligible for lung transplants in most centers.^1,2^ Here, we present the case of a 55-year-old woman who successfully underwent bilateral lung transplant after an extended ECMO period of 182 days.

## Case Presentation

**Figure 1.**
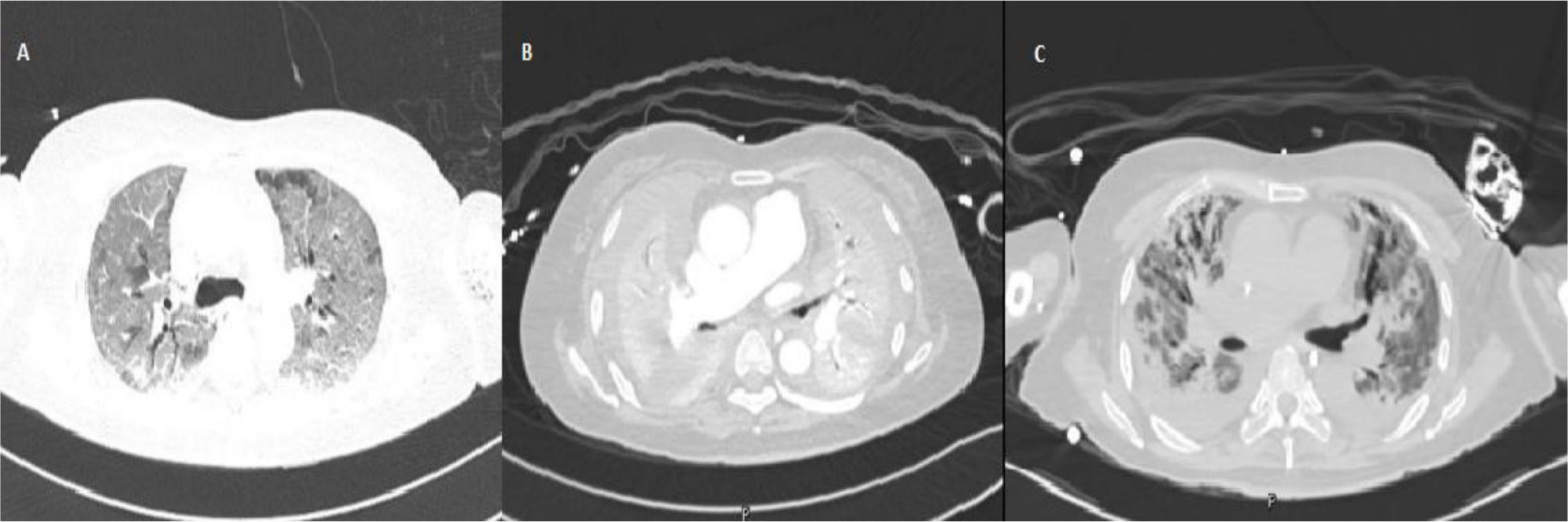
CT chest showing various phases of lung abnormalities, including bilateral ground-glass opacities (A), bilateral dense consolidations (B), and bilateral traction bronchiectasis with interstitial infiltrates (C).

A 55-year-old woman, with no prior medical history, developed an adverse skin reaction to intravenous glutathione, leading to hospitalization. Her condition worsened, resulting in severe respiratory distress. A chest CT scan revealed progressive lung abnormalities, including extensive ground glassing, bilateral thick consolidations, and interstitial infiltrates with traction bronchiectasis (Figure 1). Despite various treatments, including antibiotics, steroids, mycophenolate, rituximab, and plasma exchange, her condition deteriorated. The patient required mechanical ventilation and later ECMO support. Unfortunately, her condition did not significantly improve, and an autoimmune panel, including anti-MDA5, was negative. Due to the inability to wean off ECMO, lung transplant was proposed. After 182 days of ECMO and subsequent rehabilitation, she successfully underwent bilateral lung transplant, with pathology revealing bronchiolitis obliterans. She experienced no complications postoperatively, and ECMO was successfully weaned off within 48 hours. At three months post-transplant, she showed no transplant-related complications but faced challenges in rehabilitation.

## Discussion

Our case illustrates the extended use of ECMO as a bridge to lung transplant, prompting ethical, medical, and resource allocation considerations. Our patient’s duration of ECMO support is among the longest documented.^3^ However, her long-term prognosis is suboptimal, likely influenced by physical deconditioning from prolonged immobility despite rehabilitation during ECMO.

## Conflict of Interest

Authors have no conflict of interest to declare.
